# Anastatin Derivatives Alleviate Myocardial Ischemia-Reperfusion Injury via Antioxidative Properties

**DOI:** 10.3390/molecules26164779

**Published:** 2021-08-06

**Authors:** Ying Fu, Cai Zhao, Rengui Saxu, Chaoran Yao, Lianbo Zhao, Weida Zheng, Peng Yu, Yuou Teng

**Affiliations:** 1 China International Science and Technology Cooperation Base of Food Nutrition/Safety and Medicinal Chemistry, College of Bioengineering, Tianjin University of Science and Technology, Tianjin 300457, China; fuyingying605@163.com (Y.F.); zc20200925@mail.tust.edu.cn (C.Z.); 17320056317@163.com (R.S.); yaochaoran123@163.com (C.Y.); zhaolianbo123456@126.com (L.Z.); 2 Medical College, Yanbian University, No.977 Gongyuan Road, Yanji 133002, China; A1679709514@163.com

**Keywords:** flavonoids derivatives, H9c2 cells, hypoxia/reoxygenation, cardioprotective effects

## Abstract

(±)-Anastatins A and B are flavonoids isolated from *Anastatica hierochuntica*. In a previous study, twenty-four di- and tri-substituted novel derivatives of anastatins were designed and their preliminary antioxidant activities were evaluated. In the present study, the protective effect of myocardial ischemia-reperfusion (I/R) and the systematic antioxidant capacity of 24 derivatives were further studied. Compound **13** was the most potent among all the compounds studied, which increased the survival of H9c2 cells to 80.82%. The antioxidant capability of compound **13** was evaluated in ferric reducing antioxidant power, 2,2’-azino-bis (3-ethylbenzothiazoline-6-sulfonic acid) radical scavenging, and 2,2-diphenyl-1-picrylhydrazyl assays. It was observed that compound **13** significantly reduced infarcted areas and improved histopathological and electrocardiogram changes in rats with myocardial I/R injury. Moreover, compound **13** decreased the leakage rates of serum lactate dehydrogenase, creatine kinase, and malonyldialdehyde from rat myocardial tissues and increased the level of glutathione and superoxide dismutase activities following myocardial I/R injury in rats. Taken together, we concluded that compound **13** had potent cardioprotective effects against myocardial I/R injury both in vitro and in vivo owing to its extensive antioxidant activities.

## 1. Introduction

Ischemic heart disease (IHD) is a leading cause of death worldwide [1]. Timely restoration of blood flow can effectively improve the clinical outcome of the disease; however, reperfusion is not without risk owing to the extent of cellular damage caused by reperfusion itself. Therefore, it is extremely important to develop effective therapeutic strategies against myocardial ischemia-reperfusion (I/R) injury [2]. The mechanism of myocardial I/R injury is very complicated; it includes apoptosis, oxidative stress [3], calcium overload [4], and inflammation [5]. Oxidative stress may lead to apoptosis through various signal transduction pathways, including signal-regulated kinase (ERK), c-Jun amino-terminal kinase (JNK), activation of p53 (tumor protein p53), and p66shc variant. Moreover, excessive reactive oxygen species (ROS) production can cause irreversible damage to cell membranes and cellular molecules, such as DNA, nucleic acids, protein, lipids, and start chain reactions, further damaging myocardial tissue. Oxidative stress [6], which is caused by an imbalance between free radical formation and ROS scavenging, plays an important role in the pathogenesis of I/R injury [7]. Therefore, strategies that protect the myocardium from oxidative stress are highly considered in the management of myocardial I/R injury. Therefore, owing to their potential free radical-scavenging capabilities, antioxidant-based strategies are beneficial in the management of I/R injury.

In clinical studies, antioxidants used to reduce myocardial I/R injury are mainly divided into two types: enzymes and non-enzymes. Enzymes such as catalase and glutathione peroxidase reduce the concentration of hydrogen peroxide to prevent cell damage; non-enzymatic antioxidants such as vitamin E, vitamin C, β-carotene, etc., weaken the oxidative stress response of the tissue to reduce tissue damage and promote functional repair. However, the application of both currently has certain limitations. The large molecular mass of enzyme preparations is not conducive to absorption [8]. There are also certain problems with the stability of the non-enzymes, which leads to poor stability of drugs, hence they are easily oxidized [9]. Flavonoids, as a kind of non-enzymatic antioxidants, have been reported to possess a variety of potent properties, including antioxidant, anticancer, antiapoptotic, and cardiovascular protection [10]. They prevent the formation of highly reactive oxides and inhibit oxidative reactions by scavenging ROS, such as hydrogen radicals and peroxynitrite. Anastatins A and B [11] are flavonoids with hepatoprotective activity. Anastatins A and B are active components in *Anastatica hierochuntica* (subtype Anastatica, family Brassicaceae). Currently, there are very few reports on the chemical synthesis and structural modifications of anastatins A and B. In 2003, active compounds from *Anastatica hierochuntica* were isolated and revealed to have hepatoprotective effects against D-galactosamine-induced cytotoxicity in primary cultured mouse hepatocytes. Nakashima and Eman have also shown that the compounds exert in vitro antioxidant and anticancer activities. However, the pharmacological effects of anastatins A and B and their derivatives, as well as procedures for their syntheses, remain unclear. Therefore, we aimed to synthesize anastatins A and B and some of their derivatives and evaluate them for antioxidant activities in a hypoxia/reoxygenation (H/R) model, which is effective for studying myocardial I/R injury [12].

In a previous study, we synthesized anastatins A and B and their analogs, and evaluated their antioxidant capacities using a cell culture model of H_2_O_2_(hydrogen peroxide)-induced oxidative damage [13]. Our previous results showed that anastatins A and B have potent hepatoprotective activities that seem to be related to their antioxidant capabilities.

In the present study, anastatins A and B and 24 of their derivatives were synthesized and evaluated for antioxidant capabilities using reducing power and radical-scavenging tests. In addition, a simple H9c2 cell model of H/R and a rat model of myocardial I/R injury were used to evaluate the cardioprotective effects of the compounds.

## 2. Results

### 2.1. H/R Model Establishment

**Figure 1:**
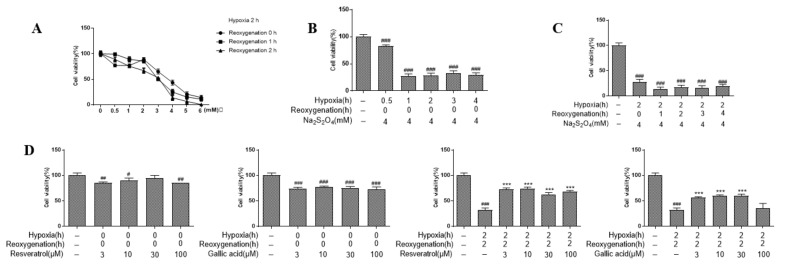
The establishment of mimic hypoxia/reoxygenation model in H9c2 cells; (**A**) Effects of different concentrations of Na_2_S_2_O_4_ on the viability of cardiomyocytes. (**B**) Effects of 4 mM Na_2_S_2_O_4_ hypoxia treatment on the viability of H9c2 cardiomyocytes at different hypoxia times. ^###^ *p* < 0.001 vs. blank. (**C**) Effects of 4 mM Na_2_S_2_O_4_ treatment on the viability of H9c2 cardiomyocytes at different reoxygenation times. ^###^ *p* < 0.001 vs. blank. (**D**) Effects of different concentrations of resveratrol/gallic acid on the survival rate of H9c2 cells. First (from left to right): cytotoxicity of resveratrol on H9c2 cells using MTT assay. Second: cytotoxicity of gallic acid to H9c2 cells using MTT assay. Third: resveratrol at different concentrations affected the survival rate of H9c2 cardiomyocytes after hypoxia for 2 h and reoxygenation for 2 h. Fourth: gallic acid at different concentrations affected the survival rate of H9c2 cardiomyocytes after hypoxia for 2 h and reoxygenation for 2 h. ^#^ *p* < 0.05; ^##^ *p* < 0.01; ^###^ *p* < 0.001 vs. blank. *** *p* < 0.001 vs. model.

The concentration of Na_2_S_2_O_4_ treatment was assessed as shown in Figure 1A, and the survival rate of H9c2 cells were reduced with increasing Na_2_S_2_O_4_ concentration. Under the 4 mM of Na_2_S_2_O_4_, cell viability decreased to less than 50% (13.7, 25, and 43.4%, respectively at the reoxygenation time of 2, 1 and 0 h) and the level of cell viability reduction was lower. Therefore, a 4 mM concentration of Na_2_S_2_O_4_ was used to mimic hypoxic treatment. Figure 1B shows that 4 mM of Na_2_S_2_O_4_ treatment for 2 h with no reoxygenation (0 h) significantly reduced cell viability, and remained steady. Under the condition of 4 mM concentration of Na_2_S_2_O_4_ for 2 h of mimicking hypoxia, 2 h of reoxygenation reduced cell survival rate to 17.37% (Figure 1C). As is clearly shown in Figure 1D, 10 μM of resveratrol reversed the reduced cell viability caused by H/R, which was more potent than the same concentration of gallic acid (74.34 vs. 60.44% for gallic acid).

As a result, the hypoxia/reoxygenation model was established by using Na_2_S_2_O_4_ at a final concentration of 4 mM for 2 h of mimicking hypoxia followed by the culture in normal DMEM (Dulbecco’s modification of Eagle’s medium)/high glucose to mimic reoxygenation injury. Resveratrol at a final concentration of 10 μM was used as a positive control.

### 2.2. Anastatins and Their Derivatives Improve the Viability of H9c2 Cells Subjected to H/R Treatment

**Figure 2:**
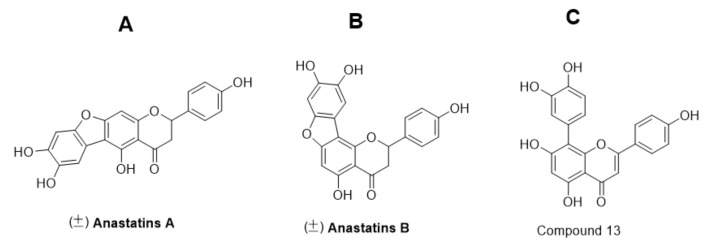
(**A**) Chemical structures of (±) anastatins A, (**B**) (±) anastatins B, and (**C**) compound **13**.

**Figure 3:**
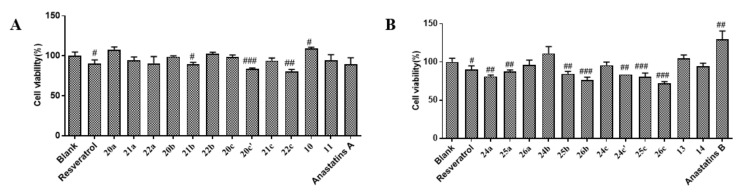
Cytotoxicity of anastatins A and B and their derivatives (10 μM) to H9c2 cardiomyocytes. (**A**) Cell viability was determined in H9c2 cells treated with anastatin A and its derivatives. (**B**) Cell viability was determined in H9c2 cells treated with anastatin B and its derivatives ^#^ *p* < 0.05; ^##^ *p* < 0.01; ^###^ *p* < 0.001 vs. blank.

**Figure 4:**
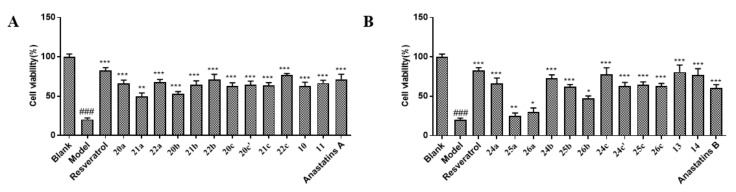
Anastatins A and B and their derivatives improved cell viability in H/R-stimulated H9c2 cells. (**A**) Anastatin A, its derivatives, and resveratrol at a final concentration of 10 μM protected H9c2 cells against H/R-induced damage. (**B**) Anastatin B, its derivatives, and resveratrol at a final concentration of 10 μM protected H9c2 cells against H/R-induced damage. ^###^ *p* < 0.001 vs. blank. * *p* < 0.05, ** *p* < 0.01, *** *p* < 0.001 vs. model.

First, we examined the effects of anastatins A and B (Figure 2A,B) and their derivatives on the viability of H9c2 cells in an MTT (3-(4,5)-dimethylthiahiazo (-z-y1)-3,5-di-phenytetrazoliumromide) assay [13]. Compounds **20a**, **21a**, **22a**, **20b**, **22b**, **20c**, **20c**′, **21c**, **22c**, **10**, and **11** were not significantly cytotoxic to H9c2 cells (Figure 3A,B, Table S2). Anastatins A and B, their derivatives, and resveratrol (positive control) reversed hypoxia-induced cell damage (Figure 4). In the model group, cell viability was decreased to 20.31%, which was increased to 82.68% by resveratrol, and anastatin A and compounds **22b**, **22c**, **24b**, **24c**, **13**, and **14** increased the cell survival rate to more than 70% (71.49, 71.00, 76.50, 73.24, 77.57, 80.82, and 77.13%, respectively). Therefore, compound **13** (Figure 2C) had the strongest protective effect among the anastatins derivatives from H/R injury and was used in the subsequent experiments.

### 2.3. Compound ***13*** Alleviates H/R-Induced Cell Damage and Oxidative Stress

**Figure 5:**
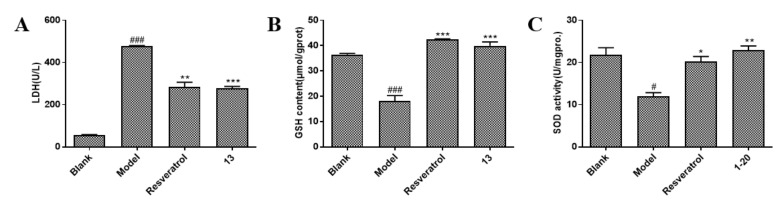
Compound **13** attenuated H/R-induced cell injury and oxidative stress in H9c2 cells. (**A**) Cell injury was determined by measuring LDH (lactate dehydrogenase) release in H9c2 cells under H/R treatment. ^###^ *p* < 0.001 vs. blank. ** *p* < 0.01, *** *p* < 0.001 vs. model. (**B**) Oxidative stress was evaluated by measuring GSH (L-Glutathione) content in cell supernatants. ^###^ *p* < 0.001 vs. blank. *** *p* < 0.001 vs. model. (**C**) Oxidative stress was also evaluated by measuring SOD (superoxide dismutase) activity in cell supernatants. ^#^ *p* < 0.05 vs. blank. * *p* < 0.05, ** *p* < 0.01 vs. model.

In order to explore the antioxidant capacity of the compound, three oxidative stress indicators, LDH (lactate dehydrogenase), GSH (L-Glutathione), and SOD (superoxide dismutase), were selected to evaluate the antioxidant capacity of compounds. LDH is the stable cytoplasmic enzyme present in myocardial cells, which is rapidly released from cell to cell culture medium upon myocardial cell membrane injury. The more serious the injury, the higher the level of LDH in the culture medium. Figure 5A demonstrated that 2 h of hypoxia followed by 2 h of reoxygenation significantly increased LDH release (478.98 U/L vs. 55.84 U/L in the blank group, *p* < 0.001), which was inhibited by resveratrol (284.07 U/L vs. the model group (H/R model), *p* < 0.01) and compound **13** (276.61 U/L vs. the model group, *p* < 0.001). GSH can act as a hydrogen donor for glutathione catalase (CAT), eliminating O^2−^ and its derivatives in the organism, thereby cells are protected from oxidant damage. Additionally, SOD can catalyze the disproportionation of superoxide anions and protect cells from damage. As shown in Figure 5B,C, compound **13** significantly increased GSH release and SOD activity (*p* < 0.01). It increased GSH level to 39.93 μmol/gprot which was higher than the level in the model group (18.2 μmol/gprot) but slightly lower than that in the resveratrol group. Additionally, it reversed H/R-induced reduction in SOD activity by increasing SOD level from 12.00 U/mgprot to 22.98 U/mgprot. These findings demonstrate that compound **13** has a protective effect on H9c2 cells subjected to H/R treatment.

### 2.4. Antioxidant Capacity of Compound ***13***

**Figure 6:**
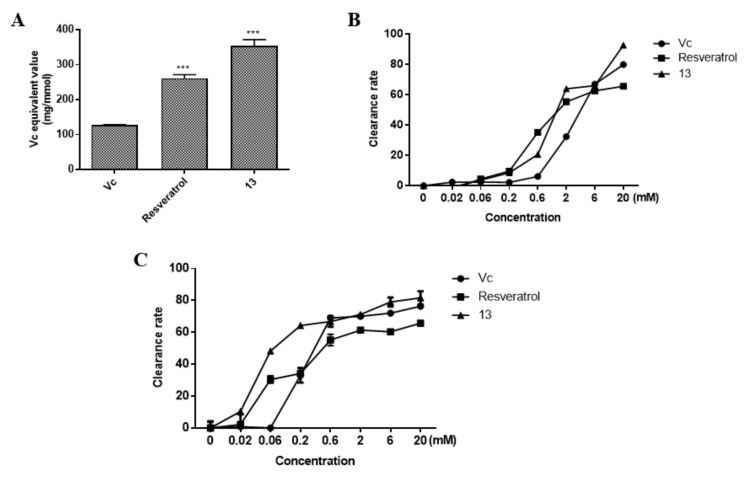
In vitro antioxidant capabilities. FRAP (**A**), ABTS (**B**), and DPPH (**C**) assay results of Vc, resveratrol, and compound **13**. *** *p* < 0.001 vs Vc group.

Vitamin C (V_C_) was used as an antioxidant control in order to evaluate the antioxidant effects of resveratrol and compound **13** intuitively. FRAP assay was used to evaluate the reducing power of the compound that can transfer ions from Fe^3^^+^ into Fe^2^^+^ to determine the antioxidant capacity of compounds. The results of the experiment showed that the reducing power of compound **13** was 354.80 mg/mmol, significantly stronger than the values obtained for the positive control Vc (127.47 mg/mmol) and resveratrol (261.91 mg/mmol) (Figure 6A). ABTS (2’-Azinobis-(3-ethylbenzthiazoline-6-sulphonate) and DPPH (2,2-Diphenyl-1-picrylhydrazyl) are stable free radicals in solvent, but when a radical scavenger is added to the solvent, the result is a reduction in the number of free radicals and the degree of reduction in the number of free radicals is directly proportional to the antioxidant capacity. Figure 6B, C show the ABTS and DPPH radical scavenging abilities of compound **13**. The results indicate that compound **13** had the strongest scavenging activity on ABTS and DPPH with EC_50_ values of 1.38 and 0.07 mM, respectively. The antioxidant capacities of the compounds tested in all three antioxidant assays followed the same trend: compound **13** > resveratrol. Therefore, the antioxidant capacity of compound **13** may be higher than those of other typical antioxidants.

### 2.5. Effects of Anastatin Derivative ***13*** on Myocardial I/R Injury in Rats

#### 2.5.1. Effects of Compound **13** on Oxidative Stress and Antioxidant Enzyme Activities

**Figure 7:**
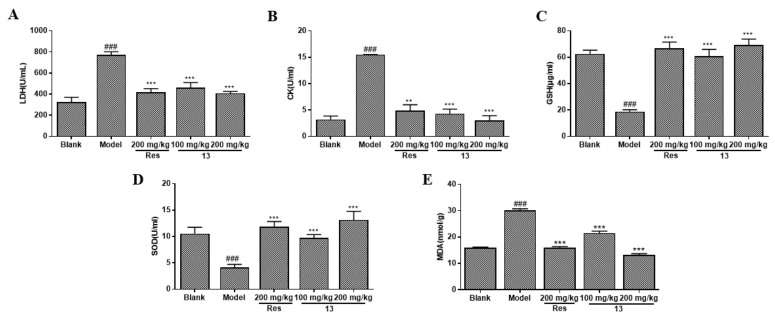
Compound **13** protected against myocardial injury in rats. Levels of LDH (**A**), CK (**B**), GSH (**C**), SOD (**D**) in serum and MDA (**E**) in myocardial homogenate in different treatment groups. ^###^ *p* < 0.001 vs. blank. ** *p* < 0.001 vs. model. *** *p* < 0.001 vs. model.

The level of myocardial injury was assessed by detecting the oxidative stress marker and antioxidant enzyme activities. The effects of compound **13** on MDA (malondialdehyde) activity in myocardial tissue, as well as the levels of LDH, CK (Creatine Kinase), GSH, and SOD in serum were assessed. As shown in Figure 7, I/R injury resulted in an increase in the levels of LDH (Figure 7A) and CK (Figure 7B) and a significant decrease in GSH (Figure 7C) and SOD activities (Figure 7D) (*p* < 0.001). The level of MDA (Figure 7E) increases after I/R injury, which is a critical diagnostic marker of I/R injury. However, pretreatment with a positive control drug and different concentrations of compound **13** significantly alleviated these injuries. LDH activity obtained for the I/R model group was 771.37 U/mL, which was remarkably decreased to 416.129 U/mL under resveratrol treatment. In the high- and low-dose 13 groups, LDH activity decreased to 408.05 and 462.81 U/mL, respectively. Similar trends were observed for other myocardial injury markers, including the level of CK, GSH, SOD, and MDA release. These results indicated that compound **13** exerted protective effects against oxidative stress-induced I/R injury in a dose-dependent manner.

#### 2.5.2. Effects of Compound **13** on Electrocardiogram (ECG) Changes and Heart Rate

**Figure 8:**
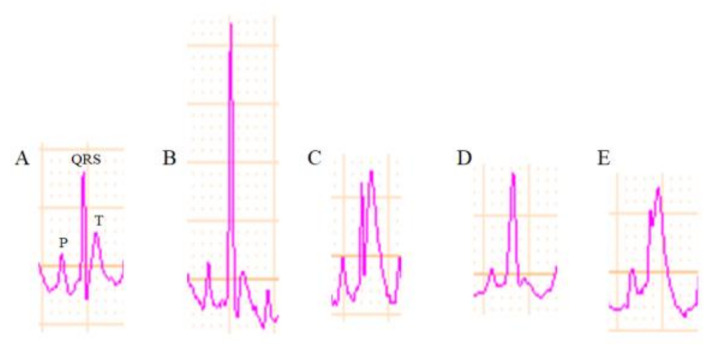
Typical myocardial I/R injury on ECG. (**A**) Normal rat ECG; (**B**) QRS amplitude increase; (**C**) QRS wave and ST wave fusion; (**D**) ST segment elevation; (**E**) ST segment inversion.

**Figure 9:**
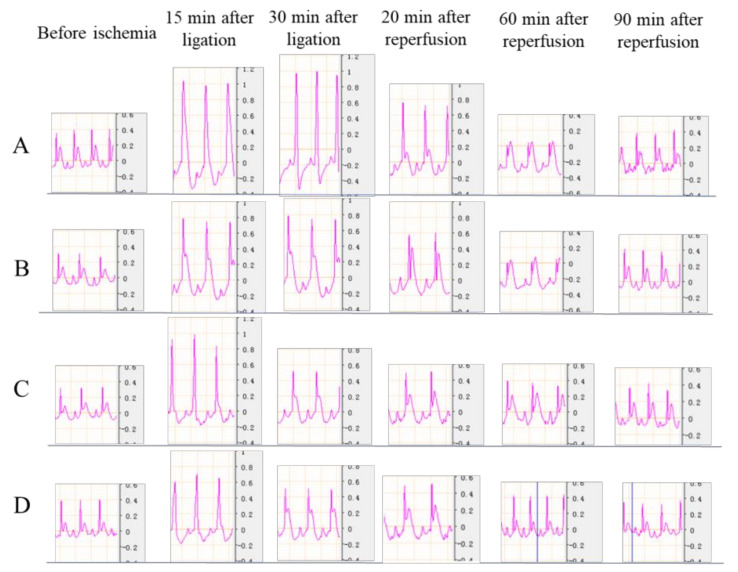
ECGs of rats with myocardial I/R injury in the model group (**A**), resveratrol group (**B**), low-dose 13 group (**C**), and high-dose 13 group (**D**).

Typical ECG (electrocardiogram) changes induced by myocardial I/R injury are shown in Figure 8. Compared with normal ECG (Figure 8A), myocardial I/R injury can cause QRS (part of electrocardiographic wave) amplitude increase (Figure 8B); QRS wave and ST wave fusion (Figure 8C); ST segment (the interval between the S wave and the T wave) elevation (Figure 8D); and ST segment inversion (Figure 8E). After 15 min of LAD ligation, QRS amplitude was significantly increased from around 0.5 mV to 1.2 mV in the model group (Figure 9A), while a lower QRS amplitude increase was observed in all of the treatment groups (from around 0.4 mV to 1, 0.8, and 0.6 mV for the resveratrol, low dose 13, and high dose 13 group, respectively) (Figure 9B–D). Additionally, a similar trend was observed at the ligation of 30 min. During the reperfusion, the QRS amplitude started to decrease; however, the QRS wave in the model group remained the highest in the whole period. After 20 min of reperfusion, the QRS amplitude in the model group reduced to about 0.8 mV, the value in the treatment group was about 0.6, 0.5, 0.5 mV for the resveratrol, low dose 13, and high dose 13 group, respectively. Overall, the treatment of resveratrol, low dose 13, and high dose 13 all reduced pathological QRS amplitude increase compared with the model group during both ischemia and the early reperfusion process, which may attributed to their myocardial protective activity.

Along with the monitoring of ECG, the changes in heart rates were detected closely. As shown in Table S3, 30 min of ligation reduced the heart rate dramatically in all groups, while no significant differences were detected between any of the treatment groups and model groups. After 2 h of reperfusion, heart rates increased but no significant changes were found among any of the groups.

#### 2.5.3. Effects of Compound **13** on Infarcted Area and Histopathological Changes

**Figure 10:**
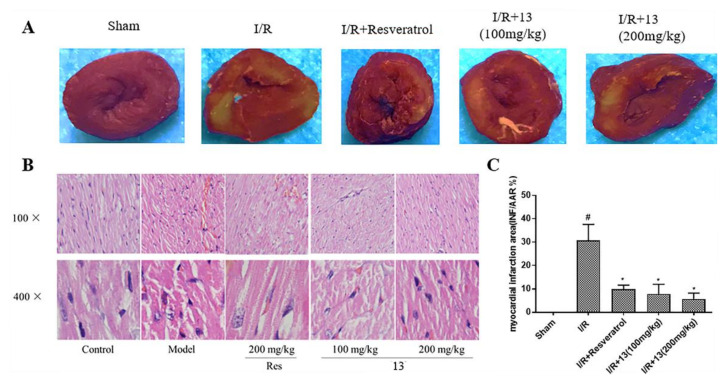
Effect of resveratrol, low-dose 13, and high-dose 13 on I/R-induced myocardial infarction. (**A**) TTC-stained images of rat heart sections from each group. (**B**) Pathological morphology of the myocardial tissue of rats with myocardial I/R injury. Heart tissues were stained with HE and visualized under a light microscope at 100× and 400× magnifications. (**C**) The percentage of infarct area in the area at risk in each group. Values were determined as means ± standard error. ^#^ *p* < 0.05 vs. sham group. * *p* < 0.05 vs. I/R group.

To evaluate the level of myocardial injury, heart tissues were stained by TTC (2,3,5-Triphenyltetrazolium chloride) and histopathological changes were detected. As shown in Figure 10, in the sham group, no changes were observed in the myocardium following TTC staining while a scale of about 30% of white infarcted areas were observed in the I/R group. In the resveratrol group and low-dose 13 group, infarcted areas were significantly diminished to around 10% and the infarcted area in the high-dose 13 group was minimized to around 5%. Figure 10 shows the result of HE (hematoxylin eosin) staining examination: enlargement of cardiac cells, partially dissolved membranes and nuclei, and inflammatory cell infiltration were observed in tissue sections from the model group. However, pretreatment with compound **13** significantly alleviated these histopathological changes. The results of TTC staining and HE staining indicated that compound **13** can reduce I/R-induced myocardial infarction in a dose-dependent manner.

## 3. Discussion

Aiming at the potential antioxidant activity of anastatins A and B, our laboratory synthesized 24 derivatives of anastatins and conducted a preliminary analysis of the cytotoxicity of compounds against PC-12 and their cytoprotective activities in H_2_O_2_-induced oxidative damage [13]. In this study, we mainly conducted the in-depth biological evaluation of 13 of the 24 derivatives with the strongest antioxidant activities.

In the present study, myocardial I/R injury was established in H9c2 cells through induction with H/R treatment. The results of the study indicated that anastatins A and B and their studied derivatives improved the viability of H9c2 cells following H/R treatment. Compound **13** was found to be the most potent among the 26 compounds studied and therefore was selected for further evaluation. The activities of LDH (a marker of cell injury), SOD, and GSH (oxidative stress markers) were detected in the supernatants of H9c2 cells following H/R treatment. We found that pretreatment with the test compounds significantly decreased LDH level and increased SOD and GSH levels in the cells. Thus, we hypothesized that anastatin derivatives possessed cardioprotective activity that may be attributed to their ability to suppress oxidative stress. The DPPH and ABTS assays are used to evaluate radical-scavenging power of the compound [14]. As a result, compound **13** had high antioxidant power, and it scavenged ABTS and DPPH at low EC_50_ values, further indicating suppression of oxidative stress. LAD ligation is a common method used to establish myocardial I/R models. By assessing the amplitude of QRS complex before ischemia, after ischemia, and after reperfusion, we found that pretreatment with compound **13** can alleviate ventricular arrhythmia significantly during ischemia and early reperfusion. Furthermore, compound **13** affected the infarction areas and histopathological changes of myocardial infarction as well. These results, along with the findings that compound **13** suppressed oxidative stress and increased the levels of antioxidant enzymes in rat serum in a dose-dependent manner, showed that compound **13** had a potent effect against myocardial injury.

In conclusion, anastatin derivatives had potent antioxidant capabilities, with compound **13** showing the highest antioxidant activity among the tested compounds. The mechanisms responsible for the effect of anastatins derivatives on oxidative stress-related signaling pathways still need to be discussed. Astaxanthin (subtype of carotenoid) can scavenge ROS and further alleviates ischemia/reperfusion injury [15]. Drugs with the potential to prevent ROS generation might be promising in oxidative stress-related diseases [4] such as cell therapy and cardiovascular diseases. Furthermore, ROS suppress the hypoxic induction of hypoxia inducible factor-1 (HIF-1) target gene expression, which is reported to have a cardioprotective effect on ischemia-reperfusion injury [16]. We hypothesize that ROS and HIF-1 regulatory protein may play a crucial role in the cardioprotective effect of compound **13**. Tournefolic acid B (TAB), which is derived from a Chinese herbal medicine, protects against myocardial I/R injury [17]. The study revealed the important roles of TAB in endoplasmic reticulum (ER) stress, apoptosis, and PI3K (phosphatidylinositol 3 kinase) /AKT (protein kinase B) pathways in the management of myocardial I/R injury. Other pathways, such as the mitogen-activated protein kinase (MAPK) pathway [18] and AMPK (Adenosine 5‘-monophosphate-activated protein kinase) signaling pathway [19], may also be involved in myocardial I/R injury. Therefore, the next step is to start with ROS and HIF-1 regulatory proteins, and use TAB-mediated pathways to explore the mechanisms by which 13 exerts an antioxidant effect and protects myocardial I/R. Moreover, the experiments at the cell level in this study should also be carried out in human cell lines.

## 4. Materials and Methods

### 4.1. Cell Culture

Rat cardiomyocyte H9c2 cell line was purchased from Nanjing Kebai Biotechnology Company (Nanjing, China). Cells were cultured in DMEM/High glucose (Hyclone, USA) and supplemented with 10% FBS (Lanzhou Minhai Biological Engineering co. LTD, Lanzhou, China) and 1% penicillin/streptomycin (Hyclone, Marlborough, MA, USA) in a humidified incobator at 37 °C with 5% CO_2_.

### 4.2. Cell Viability Assay

Cell viability was measured using the MTT assay. H9c2 cells were seeded in a 96-well plate at 1 × 10^4^ cells/well for 24 h. Afterwards, cells were treated with Anastatins A and B, and their derivatives for 48 h or pretreated with Anastatins A and B, and their derivatives followed by H/R treatment. Then, the MTT (20 μL/well) was added and cultured at 37 °C for 4 h. The absorbance was measured at 578 nm by using a microplate reader (Tecan, Infinite 50). The cell viability in the blank group was considered 100%.

### 4.3. Hypoxia/Reoxygenation (H/R) Model and Drug Administration

#### 4.3.1. H/R Model

Na_2_S_2_O_4_ was used to mimic the hypoxic condition, which reacts with [20] oxygen and reduces the oxygen tension. A variety of seven concentrations of 0.5, 1, 2, 3, 4, 5, or 6 mM were conducted to determine the optimal concentrations of Na_2_S_2_O_4_. To select optimal hypoxia time, cultures of 0.5, 1, 2, 3, or 4 h were conducted after Na_2_S_2_O_4_ solution treatment. The culture under normal DMEM/high glucose medium for 0, 1, 2, 3, or 4 h was conducted to mimic the reoxygenation time [21].

#### 4.3.2. Drug Administration

For drug administration, anastatins A and B and their derivatives (chemical structures in Table S1) were separated into two groups: anastatins A and its derivatives, and anastatins B and its derivatives. Resveratrol, which was reported to have a cardio-protective effect through its antioxidant activity, was used as a positive control. To determine the concentration of the positive control, resveratrol and another antioxidant compound, gallic acid (3–100 μM), were evaluated using MTT assay in which cells were treated for 48 h [22]. In general, H9c2 cells were pretreated with anastatins A and B and their derivatives (10 μM of final concentration) as well as 10 μM of resveratrol for 30 min before H/R treatment.

### 4.4. Antioxidant Capacity Evaluation

#### 4.4.1. Ferric Reducing Antioxidant Power (FRAP) Assay

The FRAP assay was done according to Qiyuan’s method [23]. A total of 25 μL of 1% potassium ferricyanide K_3_Fe(CN)_6_ was pipetted and 10 μL of compound **13**, vitamin c (ascorbic acid, Vc), and resveratrol (0.02–20 mM) plus 1 × PBS (pH = 6.6) were added. The mixture was kept in 50 °C water baths for 20 min followed by rapid ice cooling. Then, 25 μL of 10% trichloroacetic acid (TCA), 0.1% ferric chloride (FeCl_3_), and distilled water were added in sequence. The mixture was transferred into a 96-well plate and the absorbance was analyzed at 650 nm. Vc solution (20–200 μg/mL) was used to build a standard curve. The antioxidant capabilities were calculated from the linear calibration curve and represented as Vc equivalents.

#### 4.4.2. ABTS Method

The ABTS radical scavenging activity [24] was performed according to Sugahara et al. and Re et al. ABTS solution was diluted to adjust the absorbance spectrophotometrically at 650 of 0.70 ± 0.02 nm, and ABTS scavenging activities were measured according to the following formula, E = ((A_c__ontro__l_ − A_blank_) − (A_2_ − A_1_)/(A_control_ − A_blank_)) × 10, where A_control_ is the absorbance of the control reaction containing 130 μL ABTS^•+^ working solution and 5.5 μL sample diluent (DMSO), and A_blank_ is the absorbance of 130 μL sodium acetate buffer and 5.5 μL DMSO. A_1_ is the absorbance of 130 μL ABTS^•+^ working solution (sodium acetate buffer) and 5.5 μL of compound **13** (0.02–20 mM), and A_2_ is the absorbance of 130 μL sodium acetate buffer and 5.5 μL of compound **13** (0.02–20 mM). All mixtures were vortexed for 30 s and incubated at room temperature for 10 min followed by measuring the absorbance at 650 nm. The EC_50_ was calculated using the linear relation between the compound concentration and the probability of the percentage of ABTS inhibition.

### 4.5. Ischemia-Reperfusion Model

#### 4.5.1. Animal Care

Sprague Dawley (SD) rats were obtained from PLA Mi1itary Academy of Medical Sciences Laboratory Animal Center (Beijing, China). Fifty male SD rats weighing from 250–280 g were acclimated for one week under room temperature of 23 ± 1 °C with a 12 h light/dark cycle and food and water were provided. Rats were divided into five groups: the blank group (rats received no treatment but sham operation), the model group (rats received vehicle treatment and MI/R operation), the resveratrol group (rats received 200 mg/kg resveratrol and MI/R operation), the low dose 13 group (rats received 100 mg/kg compound **13** and MI/R operation), and the high dose 13 group (rats received 200 mg/kg compound **13** and MI/R operation). Compounds were formulated in 0.5% sodium carboxymethylcellulose and dosed as a suspension at 1 mL/kg orally once daily for seven days. All animal experiments carried out in accordance with the National Institutes of Health guide for the care and use of laboratory animals and approved by the Academic Committee of Tianjin University of Science and Technology.

#### 4.5.2. Experimental Protocol

Sprague Dawley rats were anesthetized with 20% urethan (0.8 mL/100 g) and we then performed myocardial ischemia by left anterior descending (LAD) ligation. The heart was exposed through a left thoracic incision and placed on a 4–0 silk suture to perform regional ischemia for 30 min of ischemia followed by 2 h of reperfusion. The evidence of myocardial ischemia was confirmed by ST segment elevation and QRS amplitude increase. During the experiment, rats were maintained on a ventilator DW3000 (Beijing Zhishu Duobao Biological Technology Co. Ltd, Beijing, China). Heart rate and ECGs were monitored through biological signal acquisition system MD3000 (Beijing Zhishu Duobao Biological Technology Co. Ltd, Beijing, China). After monitoring the ischemia/reperfusion process, the rats were sacrificed and blood samples and heart homogenates were used to detect myocardial injury using detective commercial kits.

#### 4.5.3. TTC Staining and HE Staining

Extent of ischemic area was quantified by triphenyl tetrazolium chloride (TTC) staining. The heart samples were frozen immediately in −20 °C for 10 min and five slices of thin 1 to 2 mm slices were made. Then, the slices were placed in 1% TTC phosphate buffer (pH = 7.0) and stained at 37 °C for 20 min and were fixed in formalin. Images were obtained using a digital camera, and the percentage of infracted area (TTC-negative) and area at risk (TTC-positive) were measured using ImageJ software [25]. For HE staining, infarcted area was excised and fixed in 10% formaldehyde and then embedded in paraffin. After that, tissues were stained with HE (hematoxylin eosin) and examined under a light microscope. Dehydrated embedded sections and sealing sections were commissioned by Tianjin YiSheng Yuan Bio-technology Company (Tianjin, China).

### 4.6. Spectrophotometric Commercial Kits

Commercial kits used in this research were purchased from Beijing Solarbio Science & Technology Co. Ltd. (Beijing, China) and Nanjing Jiancheng Bioengineering Company (Nanjing, China). In the rat serum and cell supernatant, myocardial injury marker, lactate dehydrogenase (LDH), creatine kinase (CK), glutathione (GSH) and superoxide dismutase (SOD) activities were evaluated. Malonyldialdehyde (MDA) activities were measured using rat heart homogenate. All commercial kits were conducted following the manufacturer’s instruction.

### 4.7. Statistics Process

All the experiments were repeated at least three times. The experimental data was processed using GraphPad Prism7 software, and the experimental result of each group was expressed as mean ± standard deviation ($\bar{x}$ ± s). The t-test was used to compare the statistical significance between the groups. *P* < 0.05 was recorded as statistically significant.

## Data Availability

The data presented in this study are available on request from the corresponding author.

## Supplementary Materials

The following are available online, Table S1: Compounds screened for antioxidant activity, Table S2: Effects of anastatins A and B and their derivatives on hypoxia/reoxygenation injury in H9C2 cells, Table S3: Heart rate (bpm) following LAD ligation in rats.

## Sample Availability

Samples of the compounds are available from the authors.

## References

[1] Uzdensky A.B., Demyanenko S. (2021). Histone acetylation and deacetylation in ischemic stroke. Neural Regen. Res..

[2] Li H., Yin A., Cheng Z., Feng M., Zhang H., Xu J., Wang F., Qian L. (2018). Attenuation of Na/K-ATPase/Src/ROS amplification signal pathway with pNaktide ameliorates myocardial ischemia-reperfusion injury. Int. J. Biol. Macromol..

[3] Levin R.M., Xia L., Wei W., Schuler C., Leggett R.E., Lin A.D.Y. (2017). Effects of Ganoderma Lucidum shell-broken spore on oxidative stress of the rabbit urinary bladder using an in vivo model of ischemia/reperfusion. Mol. Cell. Biochem..

[4] Tan Z., Liu H., Song X., Ling Y., He S., Yan Y., Yan J., Wang S., Wang X., Chen A. (2019). Honokiol post-treatment ameliorates myocardial ischemia/reperfusion injury by enhancing autophagic flux and reducing intracellular ROS production. Chem. Biol. Interact..

[5] Hausenloy D.J., Yellon D.M. (2013). Myocardial ischemia-reperfusion injury: A neglected therapeutic target. J. Clin. Investig..

[6] Yu C., Li D., Li Z., Yu D., Zhai G. (2021). Effect of sacubitril/valsartan on inflammation and oxidative stress in doxorubicin-induced heart failure model in rabbits. Acta Pharmaceut..

[7] Wang Y., Che J., Zhao H., Tang J., Shi G. (2018). Platycodin D inhibits oxidative stress and apoptosis in H9c2 cardiomyocytes following hypoxia/reoxygenation injury. Biochem. Biophys. Res. Commun..

[8] Pavlovic M., BNáfrádi Rouster P. (2019). Highly stable enzyme-mimicking nanocomposite of antioxidant activity. J. Colloid Interface Sci..

[9] Qi H.Z., Wangi W.Z., He J.Y. (2019). Antioxidative system of Deinococcus radiodurans. Res. Microbiol..

[10] Shao L., Shao Y., Yuan Y. (2021). Pinocembrin flavanone inhibits cell viability in PC-3 human prostate cancer by inducing cellular apoptosis, ROS production and cell cycle arrest. Acta Pharmaceut..

[11] Xiang C., Cao M., Miao A., Gao F., Li X., Pan G., Zhang W., Zhang Y., Yu P., Teng Y. (2020). Antioxidant activities of anastatin A & B derivatives and compound 38c′s protective effect in a mouse model of CCl4-induced acute liver injury. RSC Adv..

[12] Qiu R., Li W., Liu Y. (2018). MicroRNA-204 protects H9C2 cells against hypoxia/reoxygenation-induced injury through regulating SIRT1-mediated autophagy. Biomed. Pharmacother..

[13] Pan G., Li X., Zhao L., Wu M., Su C., Li X., Zhang Y., Yu P., Teng Y., Lu K. (2017). Synthesis and anti-oxidant activity evaluation of (+/-)-Anastatins A, B and their analogs. Eur. J. Med. Chem..

[14] Zhao X.J., Xing T.T., Li Y.F., Jiao B.N. (2019). Analysis of phytochemical contributors to antioxidant capacity of the peel of Chinese mandarin and orange varieties. Int. J. Food Sci. Nutr..

[15] Zuluaga M., Gueguen V., Letourneur D., Pavon-Djavid G. (2018). Astaxanthin-antioxidant impact on excessive Reactive Oxygen Species generation induced by ischemia and reperfusion injury. Chem.-Biol. Interact..

[16] Nanayakkara G., Alasmari A., Mouli S., Eldoumani H., Quindry J., McGinnis G., Fu X., Berlin A., Peters B., Zhong J. (2015). Cardioprotective HIF-1 alpha-frataxin signaling against ischemia-reperfusion injury. Am. J. Physiol. Heart C.

[17] Yu Y., Xing N., Xu X., Zhu Y., Wang S., Sun G., Sun X. (2019). Tournefolic acid B, derived from Clinopodium chinense (Benth.) Kuntze, protects against myocardial ischemia/reperfusion injury by inhibiting endoplasmic reticulum stress-regulated apoptosis via PI3K/AKT pathways. Phytomedicine.

[18] Zaha V.G., Qi D., Su K.N., Palmeri M., Lee H., Hu X., Wu X., Shulman G.I., Rabinovitch P.S., Russell R.R.I. (2016). AMPK is critical for mitochondrial function during reperfusion after myocardial ischemia. J. Mol. Cell. Cardiol..

[19] Yu D., Li M., Tian Y., Liu J., Shang J. (2015). Luteolin inhibits ROS-activated MAPK pathway in myocardial ischemia/reperfusion injury. Life Sci..

[20] Shin S., Jin Z., Ham S., Lee S., Shin D., Min Y. (2019). Effect of oxygen incorporation in amorphous molybdenum sulfide on electrochemical hydrogen evolution. Appl. Surf. Sci..

[21] Wang X., Ma S., Qi G. (2012). Effect of hypoxia-inducible factor 1-alpha on hypoxia/reoxygenation-induced apoptosis in primary neonatal rat cardiomyocytes. Biochem. Bioph. Res. Co..

[22] Goszcz K., Deakin S.J., Duthie G.G., Stewart D., Megson I.L. (2017). Bioavailable Concentrations of Delphinidin and Its Metabolite, Gallic Acid, Induce Antioxidant Protection Associated with Increased Intracellular Glutathione in Cultured Endothelial Cells. Oxid. Med. Cell. Longev..

[23] Shi Q., Chen J., Zhou Q., Lei H., Luan L., Liu X., Wu Y. (2015). Indirect identification of antioxidants in Polygalae Radix through their reaction with 2,2-dipheny1-1-picrylhydrazyl and subsequent HPLC-ESI-Q-TOF-MS/MS. Talanta.

[24] Jia Y., Wang Y., Li R., Li S., Zhang M., He C., Chen H. (2021). The structural characteristic of acidic-hydrolyzed corn silk polysaccharides and its protection on the H_2_O_2_-injured intestinal epithelial cells. Food Chem..

[25] Rameshrad M., Omidkhoda S.F., Razavi B.M., Hosseinzadeh H. (2021). Evaluating the possible role of mitochondrial ATP-sensitive potassium channels in the cardioprotective effects of morin in the isolated rat heart. Life Sci..

