# Supplementary material for: Anastatin Derivatives Alleviate Myocardial Ischemia-Reperfusion Injury via Antioxidative Properties
Source: Molecules. 2021 Aug 6;26(16):4779. doi: 10.3390/molecules26164779 (PMC8399290; doi:10.3390/molecules26164779)
Supplement: Supplementary file 1 [file molecules-26-04779-s001.zip › molecules-1278605-supplementary.pdf]

molecules

MDPI

Supplementary Materials

Anastatin Derivatives Alleviate Myocardial Ischemia-Reperfusion Injury via Antioxidative Properties

Ying Fu [1], Cai Zhao [1], Rengui Saxu [1], Chaoran Yao [1], Lianbo Zhao [1], Weida Zheng [2], Peng Yu * and Yuou Teng *

[1] China International Science and Technology Cooperation Base of Food Nutrition/Safety and Medicinal Chemistry, College of Bioengineering, Tianjin University of Science and Technology, Tianjin 300457, China; fuyingying605@163.com (Y.F.); zc20200925@mail.tust.edu.cn (C.Z.); 17320056317@163.com (R.S.); yaochaoran123@163.com (C.Y.); zhaolianbo123456@126.com (L.Z.)
[2] Medical College, Yanbian University, No.977 Gongyuan Road, Yanji 133002, China; A1679709514@163.com
* Correspondence: yupeng@tust.edu.cn (P.Y.); tyo201485@tust.edu.cn (Y.T.); Tel.: +022-60-912-562 (Y.T.)

Table S1. Compounds screened for antioxidant activity [13].

Compounds	Structures	Compounds	Structures
20a	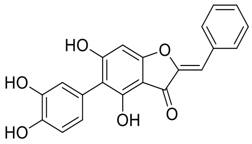	24a	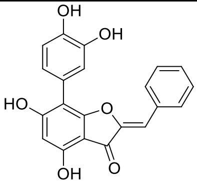
21a	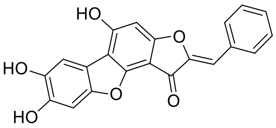	25a	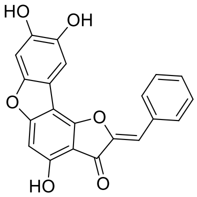
22a	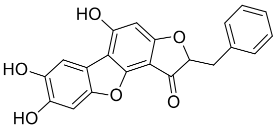	26a	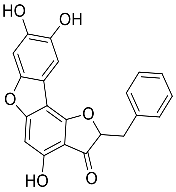
20b	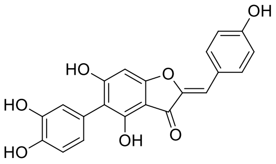	24b	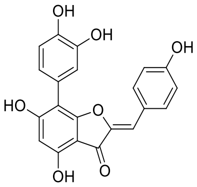
21b	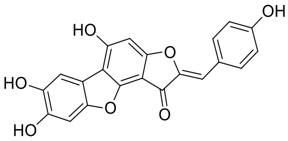	25b	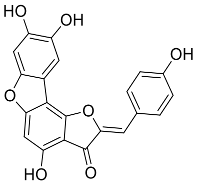
22b	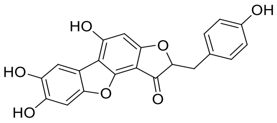	26b	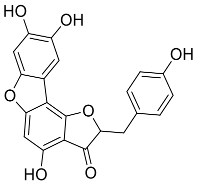

20c	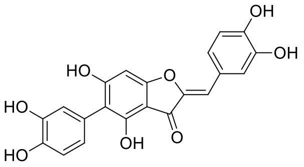	24c	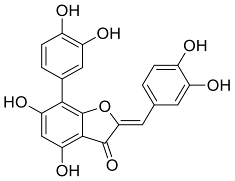
20c'	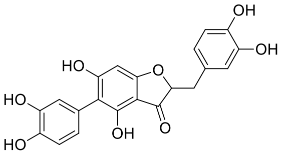	24c'	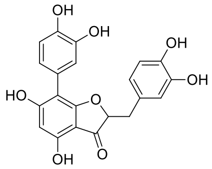
21c	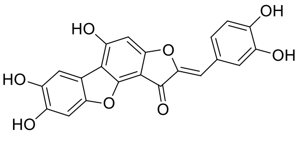	25c	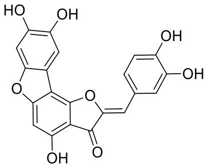
22c	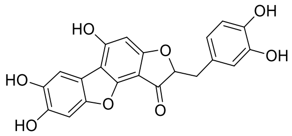	26c	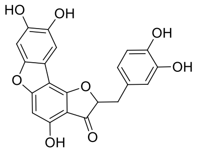
10	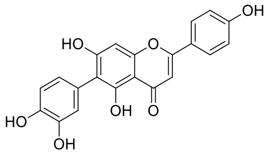	13	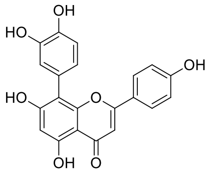
11	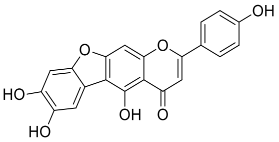	14	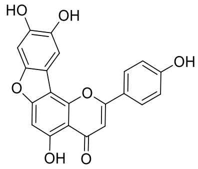
(±)Anastatins A	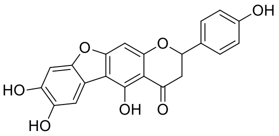	(±)Anastatins B	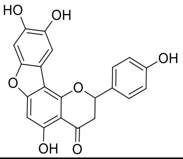

Table S2. Effects of anastatins A and B and their derivatives on hypoxia/reoxygenation injury in H9c2 cells (x̄ ± S).

**Compounds**	**$Na_2S_2O_4$ (Cell viability，%)**	**Compounds（10 μM）+$Na_2S_2O_4$ (Cell viability，%)**	**Survival rate（%）**
Blank	-	100.00±3.56	-
Model	20.31±2.06	-	-
Resveratrol	20.31±2.06	82.68±3.69	62.38
20a	20.31±2.06	66.19±4.24	45.89
21a	20.31±2.06	49.81±4.22	29.51
22a	20.31±2.06	67.78±3.39	47.48
20b	20.31±2.06	52.93±2.90	32.63
21b	20.31±2.06	64.25±5.19	43.95
22b	20.31±2.06	71.00±6.70	50.70
20c	20.31±2.06	63.03±3.95	42.73
20c'	20.31±2.06	64.33±4.83	44.03
21c	20.31±2.06	63.58±3.56	43.28

22c	20.31±2.06	76.50±2.34	56.20
10	20.31±2.06	62.88±4.81	42.58
11	20.31±2.06	66.55±3.61	46.25
Anastatins A	20.31±2.06	71.49±6.26	51.18
24a	20.31±2.06	66.19±6.96	45.88
25a	20.31±2.06	25.36±3.51	5.05
26a	20.31±2.06	29.99±5.23	9.68
24b	20.31±2.06	73.24±3.89	52.93
25b	20.31±2.06	62.35±2.57	42.04
26b	20.31±2.06	47.46±2.93	27.15
24c	20.31±2.06	77.57±8.64	57.26
24c'	20.31±2.06	62.79±4.90	42.48
25c	20.31±2.06	64.55±3.81	44.24
26c	20.31±2.06	62.78±3.55	42.47
13	20.31±2.06	80.82±8.89	60.51
14	20.31±2.06	77.13±7.90	56.82
Anastatins B	20.31±2.06	60.33±4.32	40.02

Table S3. The heart rate (bpm) following LAD ligation in rats.

	**Before ligation**	**30 min after ligation**	**2 h after reperfusion**
Model	422±10	367±9***	404±13
Resveratrol	410±16	350±24**	384±14
Low-dose 13	417±12	362±13***	391±7
High-dose 13	416±29	341±17***	376±15

All data were expressed as means ± standard error.

**$P<0.01$ different from corresponding values before ligation.

***$P<0.001$ different from corresponding values before ligation.
